# Inhibition of fibroblast growth factor receptor 3-dependent lung adenocarcinoma with a human monoclonal antibody

**DOI:** 10.1242/dmm.024760

**Published:** 2016-05-01

**Authors:** Yongjun Yin, Xiaodi Ren, Craig Smith, Qianxu Guo, Maria Malabunga, Ilhem Guernah, Yiwei Zhang, Juqun Shen, Haijun Sun, Nabil Chehab, Nick Loizos, Dale L. Ludwig, David M. Ornitz

**Affiliations:** 1 Department of Developmental Biology, Washington University School of Medicine, Saint Louis, MO 63110, USA; 2 Department of Quantitative Biology, Eli Lilly and Company, New York, NY 10016, USA; 3 Department of Cancer Angiogenesis, Eli Lilly and Company, New York, NY 10016, USA; 4 Department of Immunology, Eli Lilly and Company, New York, NY 10016, USA; 5 Department of Antibody Technology, Eli Lilly and Company, New York, NY 10016, USA; 6 Department of Bioprocess Sciences, Eli Lilly and Company, New York, NY 10016, USA

**Keywords:** Lung cancer, NSCLC, Fibroblast growth factor receptor 3, FGFR3, Adenocarcinoma, Inhibitory monoclonal antibody

## Abstract

Activating mutations in fibroblast growth factor receptor 3 (FGFR3) have been identified in multiple types of human cancer and in congenital birth defects. In human lung cancer, fibroblast growth factor 9 (FGF9), a high-affinity ligand for FGFR3, is overexpressed in 10% of primary resected non-small cell lung cancer (NSCLC) specimens. Furthermore, in a mouse model where FGF9 can be induced in lung epithelial cells, epithelial proliferation and ensuing tumorigenesis is dependent on FGFR3. To develop new customized therapies for cancers that are dependent on FGFR3 activation, we have used this mouse model to evaluate a human monoclonal antibody (D11) with specificity for the extracellular ligand-binding domain of FGFR3, that recognizes both human and mouse forms of the receptor. Here, we show that D11 effectively inhibits signaling through FGFR3 *in vitro*, inhibits the growth of FGFR3-dependent FGF9-induced lung adenocarcinoma in mice, and reduces tumor-associated morbidity. Given the potency of FGF9 in this mouse model and the absolute requirement for signaling through FGFR3, this study validates the D11 antibody as a potentially useful and effective reagent for treating human cancers or other pathologies that are dependent on activation of FGFR3.

## Abstract (precis)

**Summary:** This study validates the FGF9 lung adenocarcinoma mouse model as a tool to screen and evaluate potential therapeutics that are designed to inhibit FGF9 or its target receptor, FGFR3.

## INTRODUCTION

The fibroblast growth factor (FGF) signaling pathway is essential for organogenesis, tissue homeostasis, repair, and injury-induced angiogenesis. Aberrant activation of the FGF signaling pathway is linked to genetic disease and cancer (Dieci et al., 2013; Ellman et al., 2013; Lemieux and Hadden, 2013; Oladipupo et al., 2014; Ornitz and Itoh, 2015; Turner and Grose, 2010). The interactions of FGF receptors (FGFRs) with eighteen signaling FGF ligands regulates cell proliferation, differentiation, shape, and movement (Ornitz and Itoh, 2015). The precise cellular response to FGFR signaling, however, depends on the cell type, developmental stage and physiologic status of the organism.

Activation of the FGF signaling pathway has been implicated in animal and human cancers, where it is of considerable interest as a potential target for therapeutic intervention (Dieci et al., 2013; Liang et al., 2013; Turner and Grose, 2010). In human lung adenocarcinoma, whole genome sequencing ranked *FGFR3* among the top 25 significantly mutated genes (Imielinski et al., 2012). Additionally, increased *FGFR3* expression, mutations and gene fusions were observed in primary human NSCLC, and in cell lines derived from these tumors (Liao et al., 2013; Majewski et al., 2013); however, it has not been proven that FGFR3 activation is a driver event in these cancers. Additionally, increased expression of FGFR1, FGFR2 and FGFR3 have been implicated in the acquisition of resistance to activating mutations in the epidermal growth factor receptor (EGFR) family (Kono et al., 2009; Marek et al., 2009; Oliveras-Ferraros et al., 2012; Terai et al., 2013; Ware et al., 2013, 2010). Overexpression, activating mutations and activating gene fusions in *FGFR3* have also been identified in multiple myeloma, glioblastoma multiforme, bladder, cervical, gastric, colorectal, head and neck squamous, and germ cell-derived cancers (Dieci et al., 2013; Ornitz and Itoh, 2015; Turner and Grose, 2010). Mutations in *FGFR3* have also been identified as an escape pathway for inhibitors of B-RAF in melanoma (Yadav et al., 2012).

FGF9 is a potent ligand for FGFR3 (Hecht et al., 1995; Ornitz et al., 1996). Like *FGFR3*, *FGF9* expression has also been identified in several tumor types, including breast, prostate, endometrioid and lung (Hendrix et al., 2006; Li et al., 2008; Marek et al., 2009; Ohgino et al., 2014), suggesting an important role in tumorigenesis. Additionally, expression of *FGF9* in lung cancer was associated with poorer prognosis (Ohgino et al., 2014). To model potential oncogenic roles for FGF9, an inducible transgenic system was designed to express FGF9 in adult lung epithelium (White et al., 2006; Yin et al., 2013). Induction of FGF9 expression in adult mice resulted in the rapid conversion of cells in the bronchioalveolar duct junction into proliferative cells thought to have progenitor properties that co-express surfactant protein C (Sftpc), club cell antigen 10 (CC10, Scgb1a1) and Sca-1. Further, rapidly expanding epithelial tumors could be identified within 24-48 h of FGF9 induction. Analysis of these tumors indicated a papillary adenocarcinoma histology and expression of Sftpc, but not CC10. Moreover, genetic studies showed that the formation of these tumors was absolutely dependent on FGFR3 (Yin et al., 2013). The rapid formation of tumors and specificity for FGFR3 indicated that this model could serve as a highly stringent system to test therapeutic agents that target FGFR3 or FGF9.

In this study, we characterize a human monoclonal antibody (D11) that targets the extracellular domain of FGFR3, where it blocks ligand binding and ligand-induced signaling of both major splice variants of FGFR3. Using the FGF9-inducible mouse model, we show that treatment with the D11 monoclonal antibody can be used to prevent the initiation of tumors and slow the progression of tumors after induction of FGF9. Furthermore, treatment with D11, improved tumor-associated weight loss, reduced macrophage infiltration into lung tissue and reduced cell proliferation in the bronchioalveolar duct junction.

## RESULTS

### Characterization of a ligand-blocking anti-FGFR3 human monoclonal antibody

**Fig. 1.**
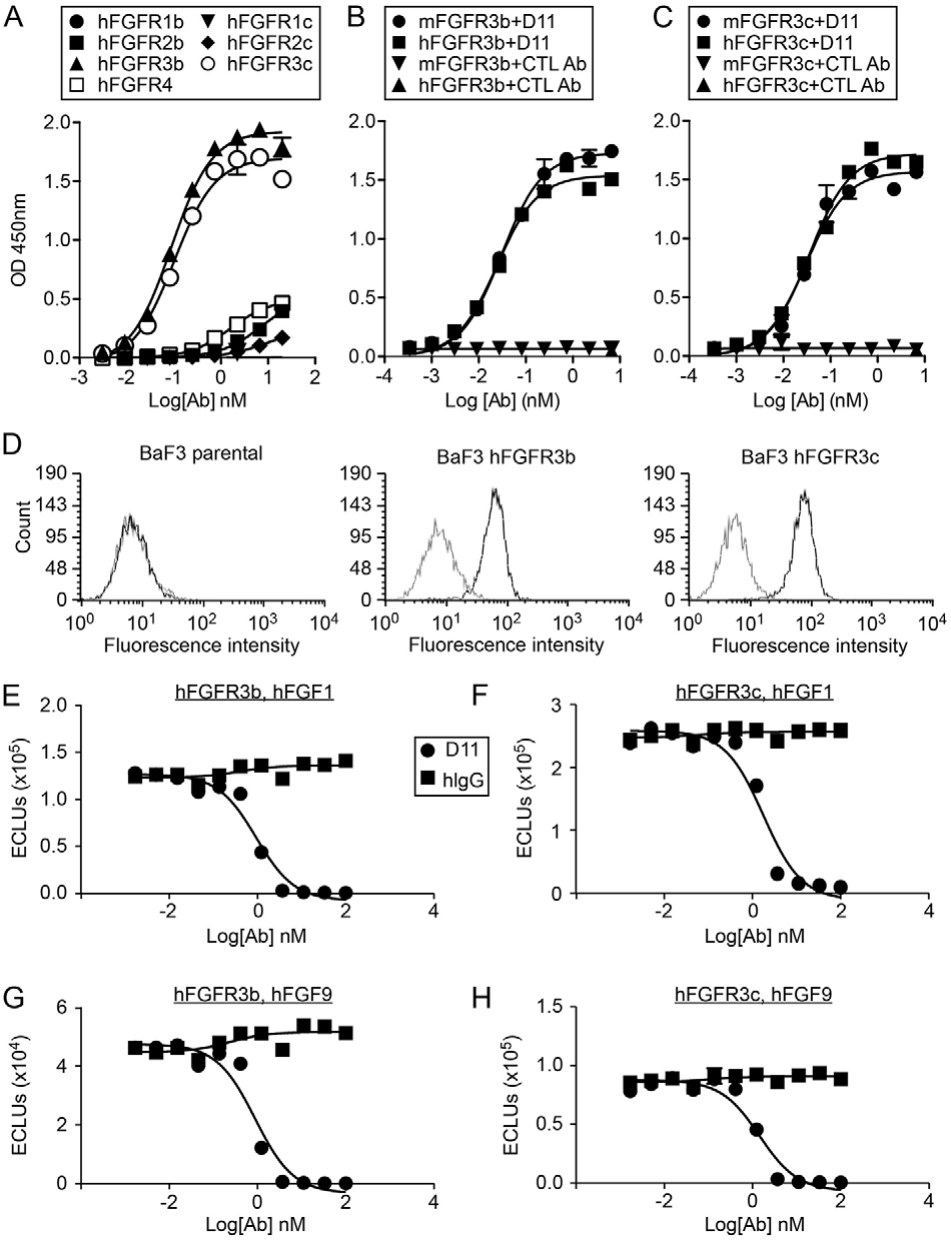
**D11 binds to FGFR3 receptor and inhibits ligand binding *in vitro*.** (A) Selective binding of D11 to FGFR3. Human FGFR1-4 Fc fusion proteins were immobilized and incubated with an increasing amount of D11 antibody. (B,C) Similar binding of D11 to human and mouse FGFR3b-Fc (B) or FGFR3c-Fc (C) fusion proteins. (D) Flow cytometry analysis of D11 binding to FGFR3b- or FGFR3c-expressing BaF3 cells. (E-H) Inhibition of FGF1 (E,F) and FGF9 (G,H) ligand binding by D11 to immobilized FGFR3b-Fc (E,G) or FGFR3c-Fc (F,H) fusion proteins. Specific binding was measured using SULFO-TAG-labeled FGF ligands in the presence of increasing amount of D11. A-D, *n*=2; E-H, *n*=3. Error bars represent s.d.

To further evaluate the role of FGFR3 in tumorigenesis and to explore the therapeutic potential of targeting this receptor, we screened a human Fab phage display library and selected an anti-hFGFR3 fully human monoclonal antibody (IMC-D11). D11 bound to human FGFR3 major splice variants FGFR3b and FGFR3c extracellular domain-Fc fusion proteins with an EC_50_ of ∼0.1 nM, and showed minimal binding to FGFR1, FGFR2, or FGFR4 extracellular domains (Fig. 1A). Additionally, D11 bound to human and mouse FGFR3b (Fig. 1B) or FGFR3c (Fig. 1C) with similar affinities. Finally, surface plasmon resonance analysis, a method for measuring protein interactions (Patching, 2014), indicated that D11 had similar binding affinity to murine, rat, cynomolgus monkey and human FGFR3b or FGFR3c (data not shown).

**Fig. 2.**
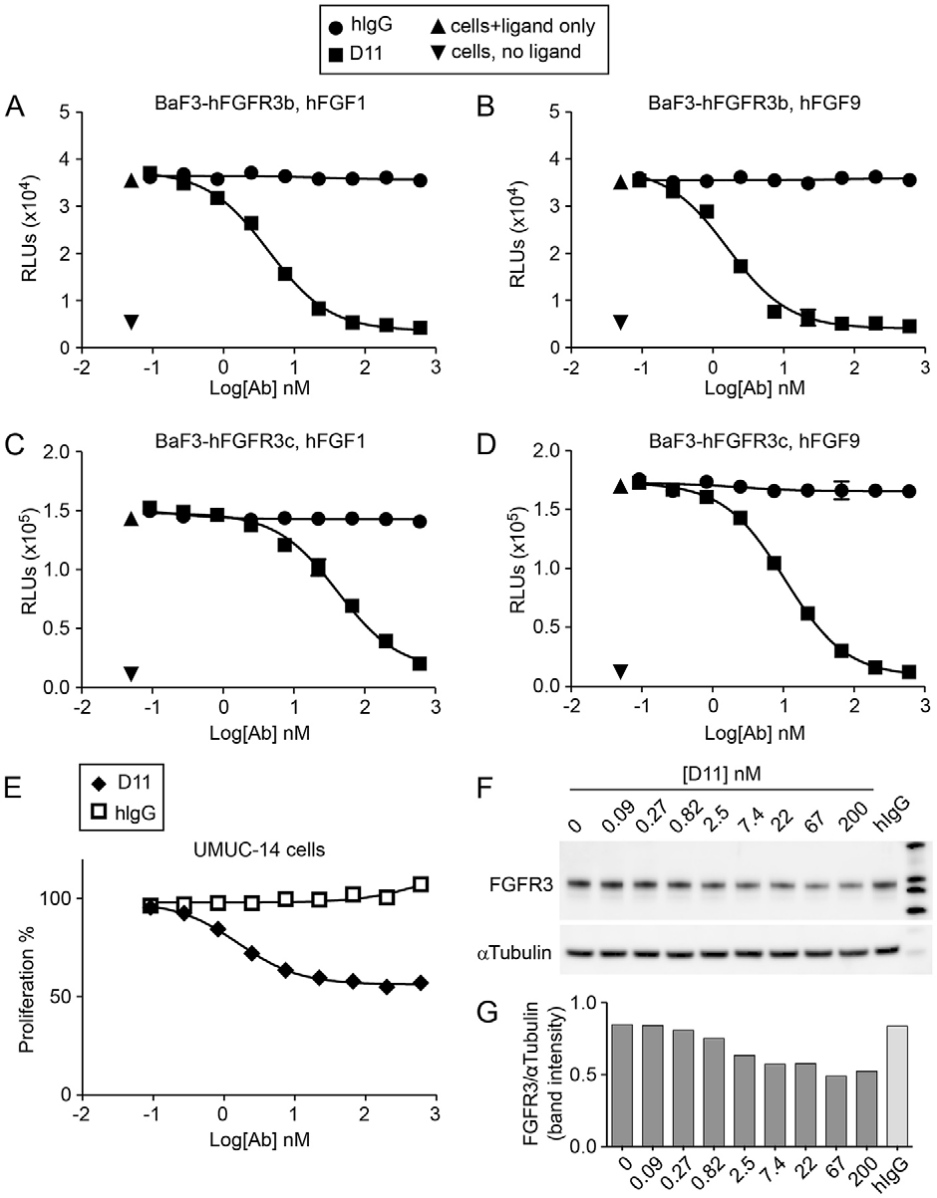
**D11 inhibits proliferation of FGFR3**-**expressing cells.** (A-D) D11 blocks ligand-dependent viability of FGFR3b- or FGFR3c-expressing BaF3 cells. Cells were cultured in growth medium without FGFs (no ligand), with 3.7 nM FGF1 or FGF9 and 15 µg/ml heparin, alone, or in combination with control antibody (hIgG) or D11. Cell viability was measured with CellTiter-Glo after 72 h incubation with antibodies. (E) D11 inhibits proliferation of bladder cancer cell line UMUC-14 harboring the FGFR3 S249C mutation. UMUC-14 cells were incubated with increasing amount of control antibody (hIgG) or D11 for 72 h. Cell viability was measured by CellTiter-Glo. (F,G) Western blot showing incubation with D11 causes FGFR3 protein loss. UMUC-14 cell lysates after 72 h incubation with control antibody (hIgG) or increasing amounts of D11 were analyzed by SDS-PAGE and probed with anti-FGFR3 and anti-tubulin antibodies (F). Bands were detected and quantified using a Fujifilm LAS-4000 Luminescence Image Analyzer (G). A-D, *n*=3; E-G, *n*=2. Error bars represent s.d.

To determine whether D11 could recognize FGFR3 splice variants expressed on the cell surface, we examined antibody interactions with BaF3 cells that stably express FGFR3b or FGFR3c. Flow cytometry analysis showed that D11 bound equally well to BaF3 cells that expressed either the FGFR3b or FGFR3c splice variants on their cell surface, but not to parental BaF3 cells that lack FGFR expression (Fig. 1D). Importantly, the binding of D11 to FGFR3 also blocked the subsequent binding of FGF1 and FGF9 to both FGFR3b and FGFR3c (Fig. 1E-H) with an IC_50_ of ∼1-2 nM. To determine whether the inhibition of ligand binding was sufficient to inhibit receptor activation, FGFR3-expressing BaF3 cells were assayed for survival and growth in response to FGF1 or FGF9 in the presence of increasing concentrations of D11. Consistent with inhibition of ligand binding, D11 also effectively inhibited the mitogenic response of FGFR3-expressing BaF3 cells to FGF1 or FGF9 with an IC_50_ ranging from 1.6 to 41.3 nM (Fig. 2A-D). D11 had no detectable agonist activity under tested conditions (data not shown).

We next examined the growth of UMUC-14 cells (Liebert et al., 1989; Miyake et al., 2007), a bladder carcinoma cell line that contains the constitutive activating point mutation, *FGFR3^S249C^*, in the presence of increasing concentrations of D11. D11 treatment significantly inhibited UMUC-14 cell growth *in vitro* in a dose-dependent manner compared with human IgG control (Fig. 2E). Importantly, D11 also caused FGFR3 receptor loss, possibly through internalization and degradation, in a dose-dependent manner in UMUC-14 cells (Fig. 2F,G). FGFR3 receptor loss mediated by D11 was also observed on multiple myeloma cell lines, OPM-2 and KMS-11 (data not shown). Thus, D11 was able to inhibit FGFR3 pathway-dependent cell proliferation through blocking ligand binding to receptors, and possibly downregulating cell surface receptors by antibody-induced receptor internalization and degradation.

### D11 inhibits FGF9-dependent lung adenocarcinoma

**Fig. 3.**
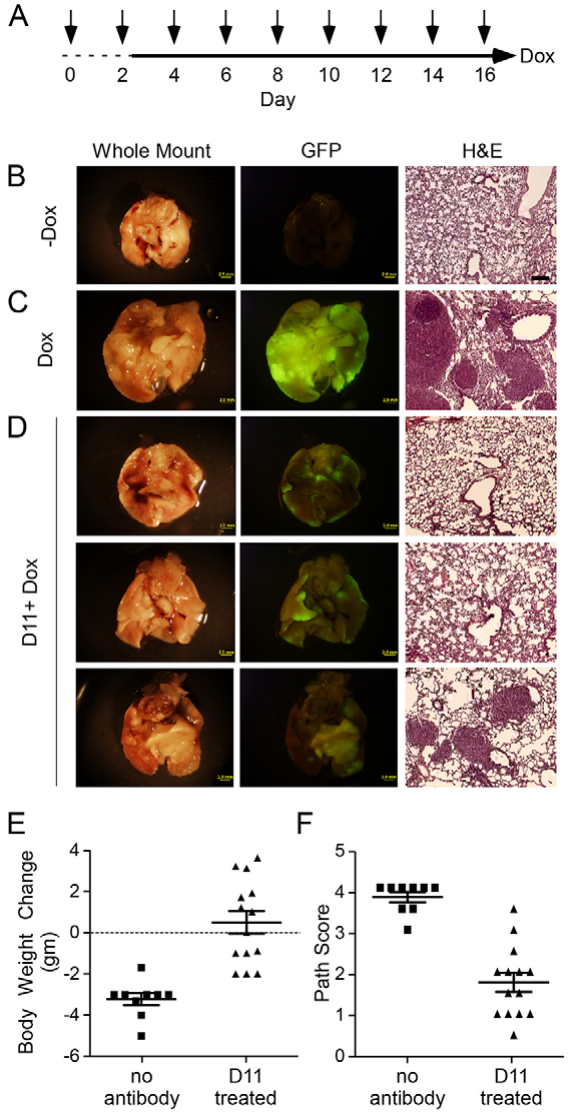
**D11 antibody prevents tumor growth in lungs induced to express FGF9.** (A) Experimental scheme for low-stringency conditions, in which mice received two D11 antibody injections (arrows) 48 h and 6 h prior to induction of FGF9 with doxycycline chow (solid line). D11 injections and doxycycline induction continued for an additional 14 days. Mice were euthanized 24 h after the last antibody injection. (B-D) Representative images of whole lungs, corresponding eGFP fluorescence, and histology, from *Sftpc-rtTA, Tre-Fgf9-Ires-eGfp* mice. (B) Uninduced control mice; (C) control mice induced with doxycycline for 14 days (1 of 9 shown); (D) experimental mice induced with doxycycline for 14 days and injected with D11 antibody (3 of 14 shown). (E) Body weight change of doxycycline-induced control (*n*=9) and D11 antibody-treated (*n*=14) mice showing reduced weight loss in D11-treated mice (*P*<0.0001). (F) Pathology score of doxycycline-induced control (*n*=9) and D11 antibody-treated (*n*=14) mice showing improved lung histology in D11 antibody-treated mice (*P*<0.0001). Scale bars: B-D whole lungs and eGFP panels, 2.0 mm; histology panels, 200 µm.

To determine whether the D11 antibody can inhibit signaling through FGFR3 *in vivo*, we employed a mouse model in which lung epithelial hyperplasia and adenomatous tumor formation is dependent on FGFR3 (Arai et al., 2015; Yin et al., 2013). The surfactant protein C–reverse tetracycline activator (*Sftpc-rtTA*) transgenic allele was used to activate the doxycycline (Dox)-responsive *Tre-Fgf9-Ires-eGfp* transgene (Tichelaar et al., 2000; White et al., 2006; Yin et al., 2013). In the absence of doxycycline, adult *Sftpc-rtTA, Tre-Fgf9-Ires-eGfp* double transgenic mice did not express GFP and their lungs were histologically normal (Fig. 3B). In response to doxycycline (provided in chow), *Fgf9* and eGFP expression were robustly induced (Fig. 3C). Within one day following doxycycline administration, small eGFP-positive nodules became visible on the lung surface. After four days of doxycycline induction, the lungs fluoresced green under UV illumination. Lung histology showed epithelial nodules with a papillary adenoma-like architecture. The receptor responsible for transducing the FGF9 signal has previously been identified as FGFR3 because induction of FGF9 on an *Fgfr3^−/−^* background did not cause epithelial hyperplasia, tumors, or an increase in epithelial proliferation (Yin et al., 2013).

**Fig. 4.**
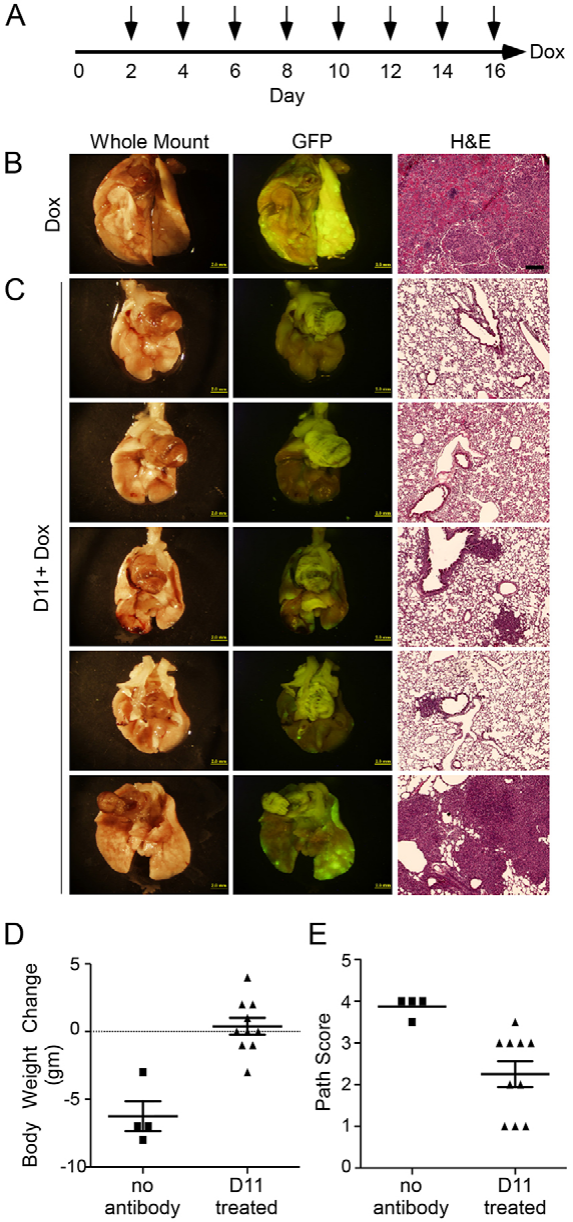
**D11 antibody inhibits tumor growth after induction of FGF9.** (A) Experimental scheme for higher stringency conditions, in which mice were induced with doxycycline chow two days before receiving the D11 antibody injection (arrows). Doxycycline induction and D11 injections continued for an additional 14 days. Mice were euthanized 24 h after the last antibody injection. (B-C) Representative images of whole lungs, corresponding eGFP fluorescence and histology, from *Sftpc-rtTA, Tre-Fgf9-Ires-eGfp* mice. (B) Control mice induced with doxycycline for 17 days (1 of 4 shown); (C) experimental mice induced with doxycycline for 12 days and injected with D11 antibody (5 of 10 shown). (D) Body weight change of doxycycline-induced control (*n*=4) and D11 antibody-treated (*n*=10) mice showing reduced weight loss in D11-treated animals (*P*<0.0002). (E) Pathology score of doxycycline-induced control (*n*=4) and D11 antibody-treated (*n*=10) mice showing improved lung histology in antibody-treated mice (*P*<0.008). Scale bars: B-C whole lungs and eGFP panels, 2.0 mm; histology panels, 200 µm.

To evaluate the ability of D11 to functionally block the FGFR3 response to induced FGF9, two experimental conditions were tested. In the lower stringency condition, the D11 antibody was injected intraperitoneally into *Sftpc-rtTA, Tre-Fgf9-Ires-eGfp* mice (40 mg/kg) beginning two days prior to induction of FGF9 with doxycycline (Fig. 3A). In the higher stringency condition, the D11 antibody was injected into *Sftpc-rtTA, Tre-Fgf9-Ires-eGfp* mice beginning two days after induction of FGF9 with doxycycline (Fig. 4A). In both cases, mice were maintained on doxycycline chow for an additional 14 days and the D11 antibody was repeatedly injected every two days. On day 17 of the experiment (24 h after the last antibody injection), mice were weighed and then euthanized. Plasma was collected to assay for circulating antibody levels and lungs were evaluated for green fluorescence and histopathology.

Control *Sftpc-rtTA, Tre-Fgf9-Ires-eGfp* mice that were not placed on doxycycline chow showed no eGFP fluorescence, and had normal lung histology (Fig. 3B) consistent with the previously observed very low background expression of the *Tre-Fgf9-Ires-eGfp* transgene. *Sftpc-rtTA, Tre-Fgf9-Ires-eGfp* mice that were given doxycycline chow for 16 days and not injected with antibody or injected with saline showed brightly fluorescent lungs with large bright green nodules (Fig. 3C). These mice showed a significant weight loss [−3.2±0.3 g (mean±s.d.), *n*=9] over the 17-day experiment. Lung histology showed large tumor nodules, in some cases occupying nearly an entire lobe. In contrast, *Sftpc-rtTA, Tre-Fgf9-Ires-eGfp* mice pre-treated with D11 antibody and injected every two days thereafter, showed improved lung histology (Fig. 3D). Importantly, these mice gained weight (0.5±0.5 g, *n*=14) over the experimental period (Fig. 3E). Plasma levels of D11 antibody were not detectable in uninjected or saline-injected mice. In D11-injected mice, the mean plasma levels, measured 24 h after the last injection, were 1124 µg/ml (range 660 to 1620 µg/ml).

To semi-quantitatively evaluate the efficacy of D11 treatment, we developed a pathology scoring system (see materials and methods and Fig. S1). In this system, a score of 0 represents normal lung and a score of 5 represents lung tissue that is filled with tumor (no normal lung). *Sftpc-rtTA, Tre-Fgf9-Ires-eGfp* mice that were induced with doxycycline and injected with saline showed an average pathology score of 3.8±0.1, *n*=9 (Fig. 3F). In contrast, mice that were treated with D11 antibody showed a significantly reduced average pathology score of 1.8±0.2, *n*=14 (*P*<0.0001 compared with controls).

The efficacy of D11 antibody treatment was next evaluated using a more stringent protocol, in which *Sftpc-rtTA, Tre-Fgf9-Ires-eGfp* mice were induced with doxycycline for two days before beginning antibody injections. After two days of doxycycline induction *Sftpc-rtTA, Tre-Fgf9-Ires-eGfp* mice showed small epithelial tumor nodules and widespread epithelial hyperplasia (Fig. S2). After a total of 17 days induction with doxycycline, control mice developed widespread adenomatous tumors (Fig. 4B) and lost significant weight (−6.3±1.1 g, *n*=4) (Fig. 4D). These mice had a mean pathology score of 3.9±0.1, *n*=4. In contrast, mice treated with D11 antibody showed improved histology (Fig. 4C), average weight gain of 0.4±0.6 g, *n*=10 (*P*<0.0001 compared with controls) (Fig. 4D), and a significantly lower average pathology score of 2.3±0.3, *n*=10 (*P*<0.008 compared with controls) (Fig. 4E). In D11-injected mice, the plasma levels of D11 antibody, measured 24 h after the last injection, were 1271 µg/ml (range 937 to 1665 µg/ml).

A third experiment was performed to examine tumor regression. Previous studies demonstrated that after doxycycline induction for up to two weeks, followed by doxycycline withdrawal for up to 18 weeks, the established tumor mass remained stable and became independent of FGF9–FGFR3 signaling (Arai et al., 2015; Yin et al., 2013). To determine whether treatment with the D11 antibody could cause tumor regression, we induced *Sftpc-rtTA, Tre-Fgf9-Ires-eGfp* mice with doxycycline for 7 days. Doxycycline chow was then replaced with normal chow and D11 antibody was injected every 2 days for 14 days. Pathology scores of control mice not injected with D11 antibody, or experimental mice injected with D11 antibody, were not different (mean pathology score: without antibody 2.5±1.4, with antibody injection 2.5±1.4), indicating that once tumors were established, inhibition of FGFR3 was not sufficient to accelerate tumor regression of these FGF9–FGFR3-independent tumors (data not shown).

### D11 inhibits the proliferation of Sftpc^+^/CC10^+^ bronchioalveolar duct junction cells

**Fig. 5.**
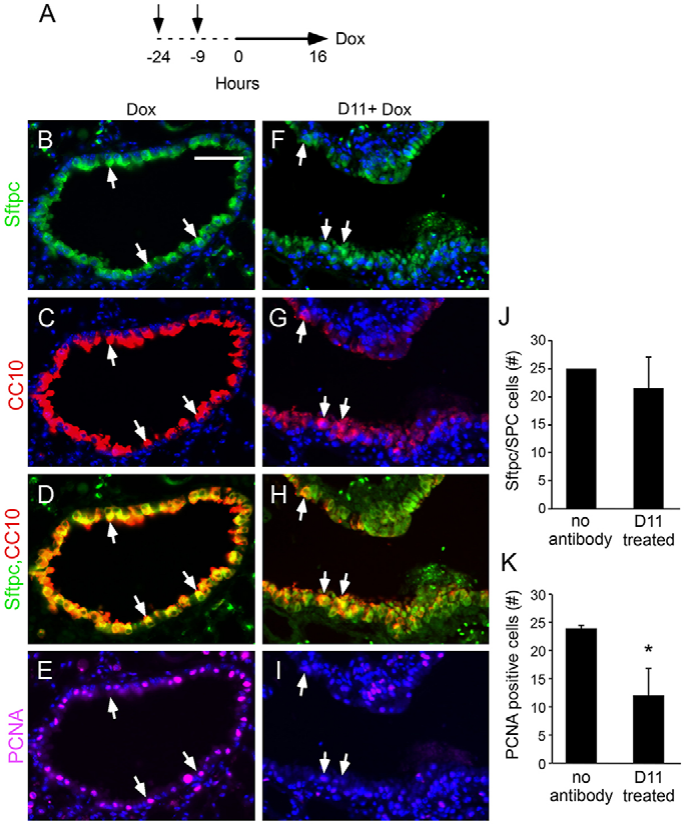
**Inhibition of Sftpc^+^/CC10^+^ double**-**positive cell proliferation by the D11 antibody.** (A) Experimental schematic. Arrows indicate D11 injections. Solid line indicates doxycycline treatment. (B-I) Immunofluorescence showing Sftpc, CC10 and PCNA expression as indicated in lung bronchioalveolar duct junction regions from *Sftpc-rtTA, Tre-Fgf9-Ires-eGfp* mice induced with doxycycline for 16 h. Control mice (B-E) were not injected and experimental mice (F-I) received two doses of D11 antibody injections prior to doxycycline induction. Arrows indicate examples of cells expressing all three markers. (J) Quantitation of the number of Sftpc^+^/CC10^+^ double-positive cells in the terminal bud region (defined as within 25 cells of the BADJ) (*n*=3 mice). (K) Quantitation of the ratio of PCNA-positive cells to Sftpc^+^/CC10^+^ double-positive cells in the terminal bud region (*n*=3 mice). Data plotted as mean±s.d., *P<0.002. Scale bar: 50 µm.

Previous studies using the *Sftpc-rtTA, Tre-Fgf9-Ires-eGfp* mouse model demonstrated that short-term (16 h) induction with doxycycline resulted in increased proliferation of Sftpc^+^/CC10^+^ cells within 25 cells of the bronchioalveolar duct junction (BADJ) (Yin et al., 2013). To determine whether D11 antibody treatment could affect proliferation or the number of Sftpc^+^/CC10^+^ cells in the bronchioalveolar duct junction region, *Sftpc-rtTA, Tre-Fgf9-Ires-eGfp* mice were injected with two doses of D11 antibody 24 h and 9 h before being placed on doxycycline chow for 16 h (Fig. 5A). After 16 h of induction, control *Sftpc-rtTA, Tre-Fgf9-Ires-eGfp* mice showed conversion of nearly all epithelial cells in the BADJ region to Sftpc^+^/CC10^+^ cells, and nearly all of these cells were positive for PCNA (Fig. 5B-E,J,K). Treatment with the D11 antibody did not affect the increase in BADJ region Sftpc^+^/CC10^+^ cells in response to induction of FGF9 (Fig. 5F-J). However, D11 antibody treatment was associated with a significant (*P*<0.002) decrease in PCNA-positive cells in the BADJ region in these mice (Fig. 5K).

### Decreased infiltration of multinucleated macrophages in D11-treated mice

**Fig. 6.**
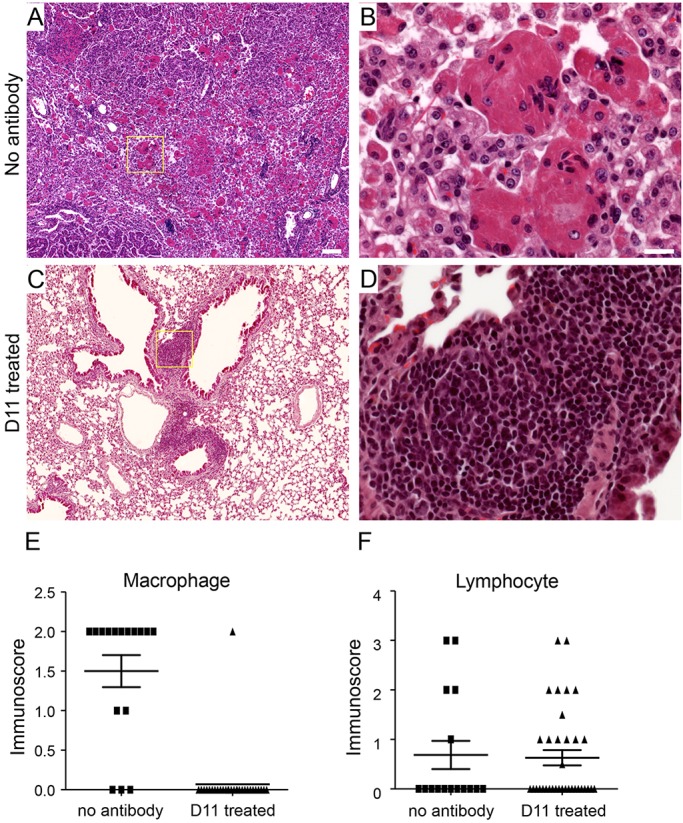
**Decreased infiltration of multinucleated macrophages in mice treated with D11 antibody.** (A,B) Lung histology from *Sftpc-rtTA, Tre-Fgf9-Ires-eGfp* mice induced with doxycycline for 16 days, but not treated with D11 antibody, showing a tumor nodule with dense infiltration of large cells referred to as multinucleated macrophages. (C,D) Lung histology from *Sftpc-rtTA, Tre-Fgf9-Ires-eGfp* mice induced with doxycycline for 16 days and treated with D11 antibody showing lack of multinucleated macrophage infiltration but occasional presence of lymphocytic nodules. Boxes in A,C are magnified in B,D. (E) Semi-quantitative immunoscore for multinucleated macrophages showing significantly reduced (*P*<0.0001) infiltration in D11-treated mice (*n*=29) compared with control (*n*=16). (F) Immunoscore for lymphocytes shows no significant difference between D11 antibody-treated (*n*=32) and untreated (*n*=16) mice. Scale bars: A, 100 µm; B, 20 µm.

Tumor induction in *Sftpc-rtTA, Tre-Fgf9-Ires-eGfp* mice is often associated with the infiltration of large multinucleated macrophages (Fig. 6A,B; Fig. S4) (Arai et al., 2015). Macrophage infiltration is evident by 24 h of induction and often resolves after several weeks, leaving behind adenomatous tumor nodules. Additionally, in some tumors (including D11-treated mice), lymphoid nodules or diffuse lymphocyte infiltration are observed throughout the lung tissue following doxycycline induction (Fig. 6C,D). The mechanism leading to immune infiltration is not known, but could result from direct signaling by FGF9 to immune cells or to inflammatory cytokine production by developing epithelial tumors.

To determine whether D11 antibody treatment affects immune cell infiltration we developed an immune score scale to evaluate the degree of immune cell infiltration (Fig. S3). Interestingly, treatment with D11 antibody significantly reduced multinucleated macrophage infiltration (Fig. 6E) but had little effect on lymphocyte infiltration (Fig. 6F; Fig. S4). This suggests that multinucleated macrophage infiltration is very sensitive to lung pathology mediated by signaling through FGFR3 whereas lymphocyte infiltration might be secondary to signals mediated by other FGF receptors.

## DISCUSSION

The future for treatment of human cancers will require redundant therapies targeted to tumor-specific pathways and the tumor microenvironment. Thus, the identification of signaling pathways required by specific tumor types and the development of therapeutic agents that effectively target these pathways will be essential to the next generation of cancer treatments. Mutation, gene rearrangements and gene amplifications that effectively activate the FGFR3 tyrosine kinase domain are currently associated with several human tumors, including adenocarcinoma of the lung, multiple myeloma, glioblastoma multiforme, nasopharyngeal, bladder, cervical, gastric, colorectal and germ cell-derived cancers (Cappellen et al., 1999; Cheng et al., 2013; Chesi et al., 2001; Ewen et al., 2013; Jang et al., 2001; Kompier et al., 2010; Ornitz and Itoh, 2015; Singh et al., 2012; Turo et al., 2015; Wang et al., 2014; Williams et al., 2013; Wu et al., 2013). Furthermore, FGF9, a high-affinity ligand for FGFR3 is expressed in a large percentage of lung, prostate, and colon cancers (Leushacke et al., 2011; Ohgino et al., 2014; Teishima et al., 2014).

In a mouse model for adenocarcinoma of the lung, activation of epithelial-expressed FGFR3 by induced expression of FGF9 results in rapid changes in the differentiation state of BADJ cells, increased proliferation of Sftpc^+^/CC10^+^ BADJ cells and type II pneumocytes, and the rapid formation of adenomatous tumor nodules (Yin et al., 2013). The rapid development of lung pathology and its complete dependence on signaling through FGFR3 makes this model ideal for testing or screening for drugs that can inhibit the FGFR3 signaling pathway *in vivo*, such as a blocking antibody to FGFR3.

D11 is a human monoclonal antibody that targets the extracellular domain of FGFR3. We show that the D11 antibody specifically recognizes human and mouse FGFR3 and has very low affinity for other FGFRs. We further show that D11 inhibits a mutant form of FGFR3 harboring a gain-of-function missense mutation, and additionally, inhibits ligand-induced activation of FGFR3. Because D11 also readily cross-reacts with mouse FGFR3, to demonstrate potential therapeutic efficacy we showed that mice given serial intraperitoneal injections of D11 over two weeks attained average plasma levels of >1000 µg/ml and showed normal weight gain and no apparent adverse effects of treatment. In a short-term treatment protocol in mice, adverse effects would most likely result from consequences of on-target inhibition of FGFR3, possible off-target interference with other physiologically active molecules, or an acute immune response. The lack of adverse effects of D11 treatment is consistent with on-target activity against FGFR3 and the knowledge that mice that congenitally lack FGFR3 are viable (Colvin et al., 1996). These studies further suggest that D11 has minimal off-target interference with other FGFRs or other non-related proteins.

The *Sftpc-rtTA, Tre-Fgf9-Ires-eGfp* mouse model is a highly stringent test for an anti-FGFR3 drug, in that FGF9 is induced throughout the lung (type II pneumocytes and distal airway epithelial cells), and the resulting lung pathology has a rapid onset characterized by acutely increased epithelial proliferation in distal airways, formation of adenomatous nodules, and inflammatory infiltration. The observation that D11, even when administered two days following FGF9 induction, has the ability to suppress FGFR3-dependent pathology *in vivo* without adverse side effects suggests that this antibody has significant therapeutic potential.

A previously developed monoclonal antibody, R3Mab, directed towards the FGFR3 extracellular domain (Qing et al., 2009) effectively blocks ligand binding and ligand-induced activation of the wild-type receptor and inhibits ligand-independent FGFR3 with activating missense mutations in the extracellular domain. R3Mab was also effective in inhibiting multiple myeloma cells containing the t(4;14)(p16.3;q32) chromosomal translocation that results in the expression of an FGFR3–MMSET fusion protein (Chesi et al., 1998; Qing et al., 2009). Other researchers have demonstrated that a single-chain anti-FGFR3 Fv fragment fused to a toxin gene product, rGel, had the ability to inhibit the growth of a xenograft bladder cancer cell line (Martinez-Torrecuadrada et al., 2008). The D11 antibody also effectively inhibits ligand binding, ligand-induced receptor activation, and activation of FGFR3 containing an activating point mutation. Furthermore, D11 causes internalization and degradation of the FGFR3 protein.

In addition to the use of antibodies to target FGFR3, small-molecule inhibitors of the FGFR tyrosine kinase domain have demonstrated efficacy with several xenograft tumor models. For example, the pan-FGFR selective tyrosine kinase inhibitors, SU5402 and PD173074, effectively blocked the growth of t(4;14)(p16.3;q32) multiple myeloma cells (Grand et al., 2004). However, no small-molecule inhibitors have been identified with specificity for FGFR3 over other FGFRs. Thus, the use of antibodies, potentially coupled to toxins or other biologically functional molecules, has the potential to provide more customized and disease-specific therapy while minimizing adverse side effects.

In addition to the role of activating FGFR3 mutations in a variety of cancers, germline-activating mutations in FGFR3 are the etiology of achondroplasia, the most common form of skeletal dwarfism in humans, and somatic activating mutations in FGFR3 cause seborrheic keratosis, a benign skin tumor, and epidermal nevi, a benign hyperplastic skin lesion (Hafner et al., 2006; Logie et al., 2005; Naski et al., 1996). Activation of the C-type natriuretic peptide (CNP) signaling pathway, which antagonizes FGFR3 signaling in chondrocytes, is being evaluated as a therapeutic for achondroplasia (Lorget et al., 2012). Antibodies that effectively inhibit FGFR3 activity *in vivo* would also be potential candidates to treat skeletal dwarfism and other pathologies associated with achondroplasia. Synergism with other activators of chondrocyte proliferation, such as CNP, might provide a more effective treatment with fewer side effects. This is important, given that treatment of achondroplasia will be required throughout much of the prepubertal growth years of affected children.

## MATERIALS AND METHODS

### Identification of a human anti-FGFR3 antibody from a phage display library

A human Fab phage display library was panned (Eli Lilly and Company, New York, NY) for anti-FGFR3IIIc antibodies using human FGFR3IIIc-Fc (R&D Systems, Minneapolis, MN; #766-FR-050) as the bait. Individual phage clones recovered after the second and third rounds of selections were examined for binding to immobilized hFGFR3IIIc/Fc and for blocking hFGFR3/FGF2 interaction by ELISA. The DNA sequences encoding the heavy-and light-chain variable genes for the selected antibody, IMC-D11 (D11; Eli Lilly and Company), were amplified by PCR and cloned into an expression vector containing human λ light-chain constant region and human γ1 heavy-chain constant region. D11 monoclonal antibody was produced from individual clones of stably transfected CHO cells.

### ELISA binding and MSD blocking assays

Various amounts of phage, Fab, or monoclonal antibody were serially diluted in 0.2% Tween 20/PBS containing 1% BSA, and added to hFGFR3IIIc-coated plates (50 µl at 1 µg/ml) and incubated at room temperature for 2 h. The bound antibodies were detected with an anti-human Fab antibody conjugated with HRP (Jackson ImmunoResearch, West Grove, PA; #109-035-097) in binding assays, or continuously probed with SULFO-TAG-labeled FGFs (Meso Scale Discovery, Rockville, MD; # R91AN-1) in blocking assays according to the supplier's instructions. 250 nM NaCl was present in ELISA binding and washing steps, and 10 µg/ml heparin was present in ligand-blocking assays. Human FGF1 (Genway Biotech Inc., San Diego, CA; #GWB-54AEB0), Human FGF9 (R&D Systems; #273-F9).

### Cell lines and proliferation assays

Murine pro-B cell line BaF3 parental cells (Mathey-Prevot et al., 1986; Palacios and Steinmetz, 1985) were maintained in RPMI-1640 (Invitrogen, Carlsbad, CA) with 10% FBS (heat inactivated, Hyclone, Logan, UT), 5 ng/ml murine IL-3 (R&D Systems); BaF3 cells stably transfected with FGFR3 were maintained in the above medium supplemented with 2 µg/ml puromycin (Sigma-Aldrich, St. Louis, MO). Cell lines were confirmed to be IL3-dependent and puromycin-resistant. No further authentication was carried out on these cells. For flow cytometry, BaF3 cells were incubated with 10 µg/ml D11 and 1:200 dilution of secondary anti-hIgG-PE (Jackson ImmunoResearch: #109-116-088). After washing, cells were analyzed on a Guava EasyCyte Plus flow cytometry system (Millipore, Billerica, MA). For IMC-D11-blocking BaF3/FGFR3 cell proliferation assays, 25,000 cells/well were seeded in 96-well plates. After incubation with serially diluted D11 for 1 h, FGF ligands were added to 3.7 nM final concentration for 72 h. Cell viability was determined using the CellTiter-Glo Luminescent Cell Viability Assay (Promega, Madison, WI).

UMUC-14 cells (Liebert et al., 1989), obtained from MD Anderson Cancer Center, were maintained in DMEM medium (ThermoFisher, Invitrogen, Grand Island, NY) supplemented with 10% FBS (Hyclone) under conditions of 5% CO_2_ at 37°C. The mutation in *FGFR3* was confirmed by PCR amplification and sequencing. No further authentication was carried out on these cells. For D11-blocking UMUC-14 cell proliferation assay, cells were incubated with 1:3 serial-diluted D11 from 200 nM and/or 200 nM human IgG control antibody (Equitech-Bio, Kerrville, TX; #SLH56) for 72 h. Cell viability was determined using the CellTiter-Glo Luminescent Cell Viability Assay (Promega, Madison, WI).

### Western blot

Cell lysates (15 µg) from UMUC-14 cells were subjected to SDS-PAGE followed by western blot. Blots were probed with anti-FGFR3 antibody (1:2,000; Sigma-Aldrich; #F0425) or anti-tubulin antibody (1:2,000; Cell Signaling Technology, Danvers, MA; #2125), and secondary anti-Rabbit IgG HRP antibody (1:10,000; Jackson ImmunoResearch; #111-035-144). Bands were detected and quantified using a Fujifilm LAS-4000 Luminescence Image Analyzer (GE Healthcare Life Sciences, Pittsburg, PA).

### Detecting mouse plasma IMC-D11 antibody

Serially diluted plasma samples or purified D11 antibodies were added to goat anti-human IgG-coated plates, and detected with an HRP-conjugated goat anti-human IgG Fc γ-specific antibody (1:10,000; Jackson ImmunoResearch; #109-035-098).

### Mice

The *Sftpc-rtTA, Tre-Fgf9-Ires-eGfp* mouse strain has been previously described (Perl et al., 2002; Tichelaar et al., 2000; White et al., 2006; Yin et al., 2013, 2008). *Sftpc-rtTA, Tre-Fgf9-Ires-eGfp* mice were maintained on an inbred FVB genetic background. All mice were housed in a pathogen-free animal facility under the veterinary care of the Department of Comparative Medicine at Washington University School of Medicine, and used at the age of six-to-twelve weeks. All protocols were approved by the Washington University Animal Studies Committee and were performed in accordance with the Animal Welfare Act and the Guide for the Care and Use of Laboratory Animals.

### Transgene induction and D11 antibody injection

Doxycycline (Dox) diet was purchased from Bio-Serv Inc. (200 mg/kg green pellets, #S3888). Mice were fasted for 6 h prior to providing doxycycline chow at 6 pm on the first day of induction. Doxycycline chow was provided *ad libitum* throughout the experiment. The D11 antibody was diluted with 1× PBS and injected intraperitoneally at a dose of 40 mg/kg (∼0.5 ml/mouse). Control mice were either not injected or injected with 0.5 ml of 0.9% saline. Body weight was measured before induction and on the day of necropsy.

### Lung pathology, histology, and immunohistochemistry

Mice were anesthetized with KXA (31 mg/kg ketamine, 6 mg/kg xylazine, 1 mg/kg acepromazine) and transcardially perfused with a vascular rinse of 0.9% NaCl followed by 10% neutral buffered formalin (VWR International; #89370-094). Lungs were dissected and photographed under bright-light illumination and under UV light using an Olympus SZX12-ILLD100 dissecting microscope (Olympus Optical Co. Ltd). Tissues were postfixed in 10% phosphate buffered formalin overnight at 4°C. All five lobes were separated, embedded in paraffin and sectioned using standard procedures. For histology, slides were stained with hematoxylin and eosin (H&E). For immunofluorescence staining, sections were rehydrated. Antigen retrieval was achieved by boiling at 121°C for 15 min in 10 mM citrate buffer followed by gradual cooling to room temperature. Sections were incubated overnight at 4°C with the primary antibodies, and after incubation with the primary antibodies, appropriate Alexa Fluor-coupled secondary antibodies (Thermo Fisher; #A21206, #A21203, #A21447) were applied at a 1:200 dilution. Sections were photographed on an ApoTome fluorescence microscope (Zeiss Inc). The following primary antibodies were used for staining: CC10 (*Scgb1a1,* sc-9772; Santa Cruz Biotechnology Inc.; 1:200); pro-SP-C (SftpC, AB3786; Millipore; 1:2000); PCNA (sc-56; Santa Cruz Biotechnology Inc.; 1:100); CD45 (ab10558; Abcam, Cambridge, MA; 1:200). For the CD45 antibody, immunostaining was detected using the Histostain-SP Broad Spectrum (DAB) kit (Thermo Fisher; #95-9643).

For quantitation of cell number, multiple optical sections were scored manually to distinguish cell boundaries and identify the bronchioalveolar duct junction. Sfptc- and CC10-positive cells were counted within 25 cells of the bronchioalveolar duct junction as described (Yin et al., 2013). Three different whole-lung longitudinal sections containing the main axial bronchi were scored for each mouse.

### Pathology and immunology scores

We developed a histopathological scoring system (Fig. S1 and S3) to provide semi-quantitative analysis of the extent of tumor growth and inflammatory infiltrates (Gibson-Corley et al., 2013). For lung tumorigenesis, we based this scoring system in part on the Ashcroft scoring system, which is commonly used to evaluate the extent of lung fibrosis (Ashcroft et al., 1988; Guzy et al., 2015).

For each mouse sample, 2-3 H&E slides were selected from different depths of the tissue, separated by at least 50 µm. Three to five lobes were viewed for each mouse. Two persons scored the slides and were blind to the identity of the mouse or the treatment. The pathology score was defined as: 0, normal lung tissue; 1, alveolar or ductal hyperplasia; 2, 1-3 tumor nodules in at least one lobe; 3, many tumor nodules in multiple lobes; 4, solid tumor in at least one lobe; 5, solid tumor, no normal lung (Fig. S1).

The immunology score was defined as: 0, no inflammation; 1-L, diffuse lymphocyte infiltration, no nodules; 2-L, peribrochiolar lymphocyte clusters, <4 clusters in one lobe with >10 cells per cluster; 3-L, peribrochiolar lymphocyte clusters, ≥4 clusters in one lobe with >10 cells per cluster; 1-M, diffuse multinucleated macrophage infiltration; 2-M, dense multinucleated macrophage infiltration (Fig. S3).

### Statistical analysis

All data are expressed as mean±standard derivation (s.d.), Student's *t*-test values indicate statistical significance for comparable tissues, *P* values were obtained using GraphPad Prism and Microsoft Excel software.
